# Enhanced antitumor effects of follicle-stimulating hormone receptor-mediated hexokinase-2 depletion on ovarian cancer mediated by a shift in glucose metabolism

**DOI:** 10.1186/s12951-020-00720-4

**Published:** 2020-11-07

**Authors:** Meng Zhang, Qiyu Liu, Mingxing Zhang, Cong Cao, Xiaoxia Liu, Mengyu Zhang, Guiling Li, Congjian Xu, Xiaoyan Zhang

**Affiliations:** 1 Obstetrics and Gynecology Hospital, Fudan University, Shanghai, 200011 China; 2 School of Materials Science & Engineering, Zhejiang Sci-Tech University, Hangzhou, 310018 China; 3 Shanghai Key Laboratory of Female Reproductive Endocrine Related Diseases, Shanghai, 200011 China

**Keywords:** Ovarian carcinoma, Hexokinase-2, Targeted therapy, Chemoresistance, Follicle-stimulating hormone

## Abstract

**Background:**

Most cancers favor glycolytic-based glucose metabolism. Hexokinase-2 (HK2), the first glycolytic rate-limiting enzyme, shows limited expression in normal adult tissues but is overexpressed in many tumor tissues, including ovarian cancer. HK2 has been shown to be correlated with the progression and chemoresistance of ovarian cancer and could be a therapeutic target. However, the systemic toxicity of HK2 inhibitors has limited their clinical use. Since follicle-stimulating hormone (FSH) receptor (FSHR) is overexpressed in ovarian cancer but not in nonovarian healthy tissues, we designed FSHR-mediated nanocarriers for HK2 shRNA delivery to increase tumor specificity and decrease toxicity.

**Results:**

HK2 shRNA was encapsulated in a polyethylene glycol-polyethylenimine copolymer modified with the FSH β 33–53 or retro-inverso FSH β 33–53 peptide. The nanoparticle complex with FSH peptides modification effectively depleted HK2 expression and facilitated a shift towards oxidative glucose metabolism, with evidence of increased oxygen consumption rates, decreased extracellular acidification rates, and decreased extracellular lactate and glucose consumption in A2780 ovarian cancer cells and cisplatin-resistant A2780CP counterpart cells. Consequently, cell proliferation, invasion and migration were significantly inhibited, and tumor growth was suppressed even in cisplatin-resistant ovarian cancer. No obvious systemic toxicity was observed in mice. Moreover, the nanoparticle complex modified with retro-inverso FSH peptides exhibited the strongest antitumor effects and effectively improved cisplatin sensitivity by regulating cisplatin transport proteins and increasing apoptosis through the mitochondrial pathway.

**Conclusions:**

These results established HK2 as an effective therapeutic target even for cisplatin-resistant ovarian cancer and suggested a promising targeted therapeutic approach.

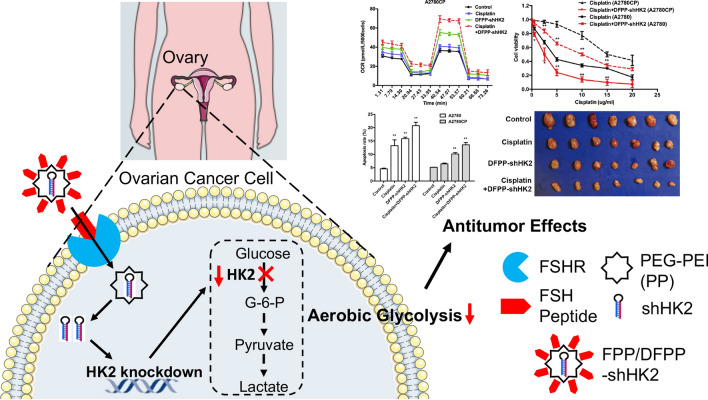

## Background

Ovarian cancer is the leading cause of gynecological malignancy-associated deaths worldwide [1]. Over 80% of patients are diagnosed with advanced-stage disease, and patients have a 10-year survival of only 15% [2]. The standard treatment for ovarian cancer is cytoreductive surgery followed by platinum-based chemotherapy. However, chemoresistance occurs in 80–90% of patients with advanced-stage disease [3]. The median survival from the time of the onset of platinum resistance is only 1 year [4]. There is a critical need to develop therapeutic strategies to overcome chemoresistance.

Metabolic reprogramming is a common feature of cancer. The Warburg effect, which is also known as aerobic glycolysis, is characterized by the enhanced conversion of glucose to lactate in cancer cells despite normal oxygen conditions [5]. Mounting evidence has suggested that metabolic shifts are related to tumor progression and chemoresistance [6–8]. Hexokinases (HKs) are the first rate-limiting enzymes in the glycolysis pathway and convert glucose to glucose-6-phosphate (G6P). Among HK family members, the expression of HK2 is relatively limited in normal adult tissues, including skeletal and adipose tissues, and HK2 is more catalytically efficient [9]. Moreover, HK2 is highly expressed in many tumor tissues and could be a potential target for antitumor therapy based on the promising results of preclinical studies [9–14].

HK2 overexpression has been observed in high-grade and advanced ovarian cancer and is correlated with early recurrence and short progression-free survival in ovarian cancer patients [15–17]. Inhibition or knockdown of HK2 significantly attenuated tumor growth and the dissemination of ovarian cancer xenografts [13, 17]. It is of note that HK2 overexpression has been associated with chemoresistance in ovarian cancer and is an independent risk factor [15]. However, the roles of HK2 inhibition in chemoresistant ovarian cancer need to be further elucidated.

Currently, substantial attempts have been made to develop HK2-inhibitory antitumor agents [18]. However, limited clinical success has been achieved. The reasons for this could be the nonspecific role of small molecular inhibitors of the HK2 enzyme and the presence of conserved pathways in both normal and cancer cells. 2-Deoxyglucose (2-DG), which is a small molecular inhibitor of HK2, had obvious anti-proliferative effects in preclinical studies. In clinical studies, it showed toxicity associated with hypoglycemia symptoms, and lower doses were insufficient to inhibit tumor progression [8]. Thus, the selectivity of HK2 inhibitors is crucial for their clinical use.

RNA interference (RNAi) has been widely used as a tool for the specific knockdown of gene expression. However, the delivery of RNAi drugs still requires additional investigation. The key is to improve the tissue specificity of RNAi so that it can be selectively delivered into cancer cells. Delivery systems based on nanocarriers have been developed to aid intracellular delivery of RNAi drugs and have shown promising results in antitumor therapy [19, 20]. Polyethylene glycol (PEG)-polyethylenimine (PEI) copolymers have been identified as suitable carriers because of their high transfection efficiency and improved colloidal stability and blood compatibility [21].

Furthermore, since tumor cells express specific surface markers that distinguish them from normal cells, targeting moieties, including polypeptides and antibodies, are often conjugated with nanocarriers to achieve tumor-specific drug delivery [22]. An ideal target for ovarian cancer is follicle-stimulating hormone receptor (FSHR) because FSHR expression is mainly limited to ovary tissues and is overexpressed in ovarian cancer [23–27]. Using a follicle-stimulating hormone (FSH) peptide as a targeting moiety, specific drug delivery could be achieved. We previously developed FSH peptide-modified nanoparticle carriers to increase drug uptake and decrease side effects in ovarian cancer [25, 28]. In contrast to nontargeted nanoparticles, FSH peptide-modified nanoparticles showed selective drug accumulation and enhanced antitumor effects. This drug delivery strategy could be used for the selective inhibition of HK2 in ovarian cancer.

Here, we developed FSH peptide- and retro-inverso FSH peptide-conjugated PEG-PEI copolymers loaded with HK2 shRNA to selectively suppress HK2 expression in ovarian cancer. We further evaluated their effects on the glucose metabolism pattern, reversal of cisplatin resistance, and antitumor effects in vitro and in vivo.

## Results and discussion

### Expression of FSHR and HK2 in A2780 and A2780CP ovarian cancer cells

**Fig. 1:**
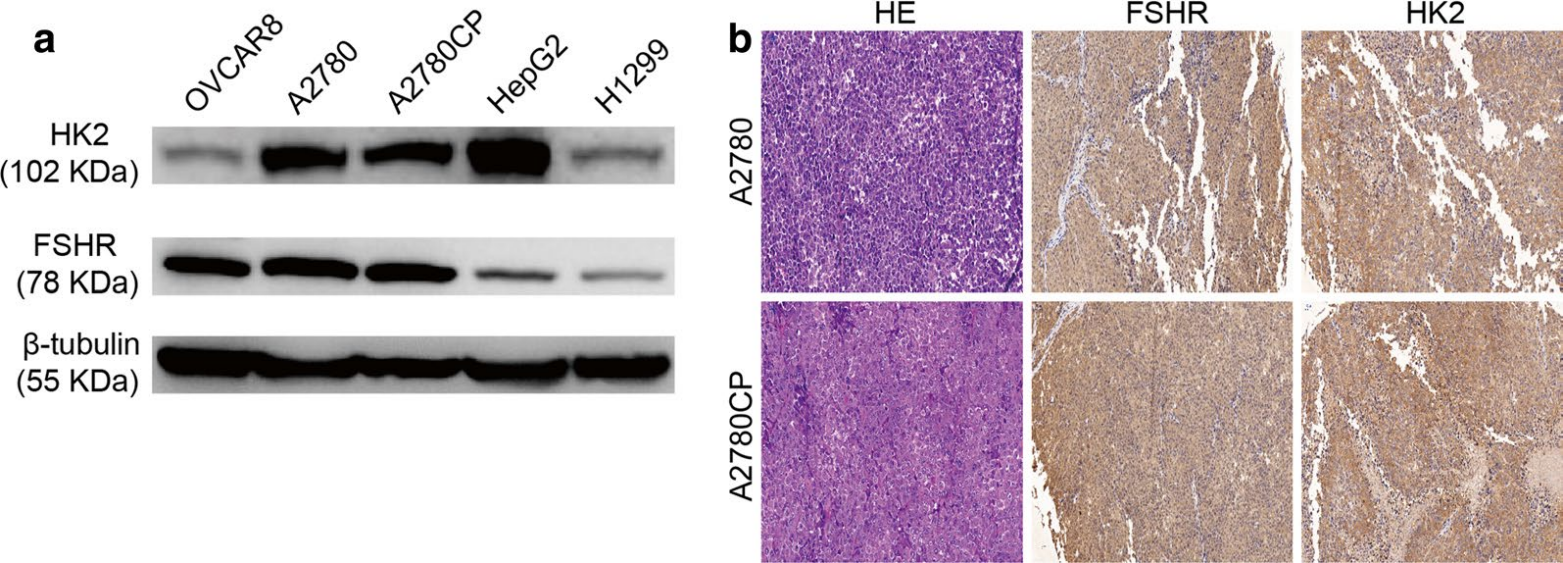
Expression of FSHR and HK2 in cancer cell lines. **a** FSHR and HK2 expression in ovarian cancer A2780, A2780CP and OVCAR8 cells, lung cancer H1299 cells, and liver cancer HepG2 cells were detected by western blotting. **b** FSHR and HK2 expression in A2780 and A2780CP tumor xenografts of nude mice detected by immunohistochemistry (200X)

To screen the appropriate cell lines, we detected the expression of FSHR and HK2 in A2780, A2780CP (cisplatin-resistant counterpart of A2780) human ovarian cancer cells and other control cancer cell lines. As shown in Fig. 1a, A2780 and A2780CP cells showed high expression levels of FSHR and HK2; the ovarian cancer cell line OVCAR8 and lung cancer cell line H1299 showed low expression levels of HK2; H1299 also showed low expression level of FSHR. Thus, H1299 and OVCAR8 cells were used as comparisons. FSHR and HK2 expression were also detected in the tumor xenografts of nude mice by immunohistochemistry. The A2780 and A2780CP tumor xenografts both expressed FSHR and HK2 protein (Fig. 1b). We analyzed the FSHR expression in various normal tissues from the GTEx Portal database (Figure S1). Higher expression of FSHR mRNA was observed in ovary than in other healthy tissues, suggesting that FSHR is an ideal target for ovarian cancer-specific drug delivery system.

### Preparation and characterization of nanoparticle complexes

**Fig. 2:**
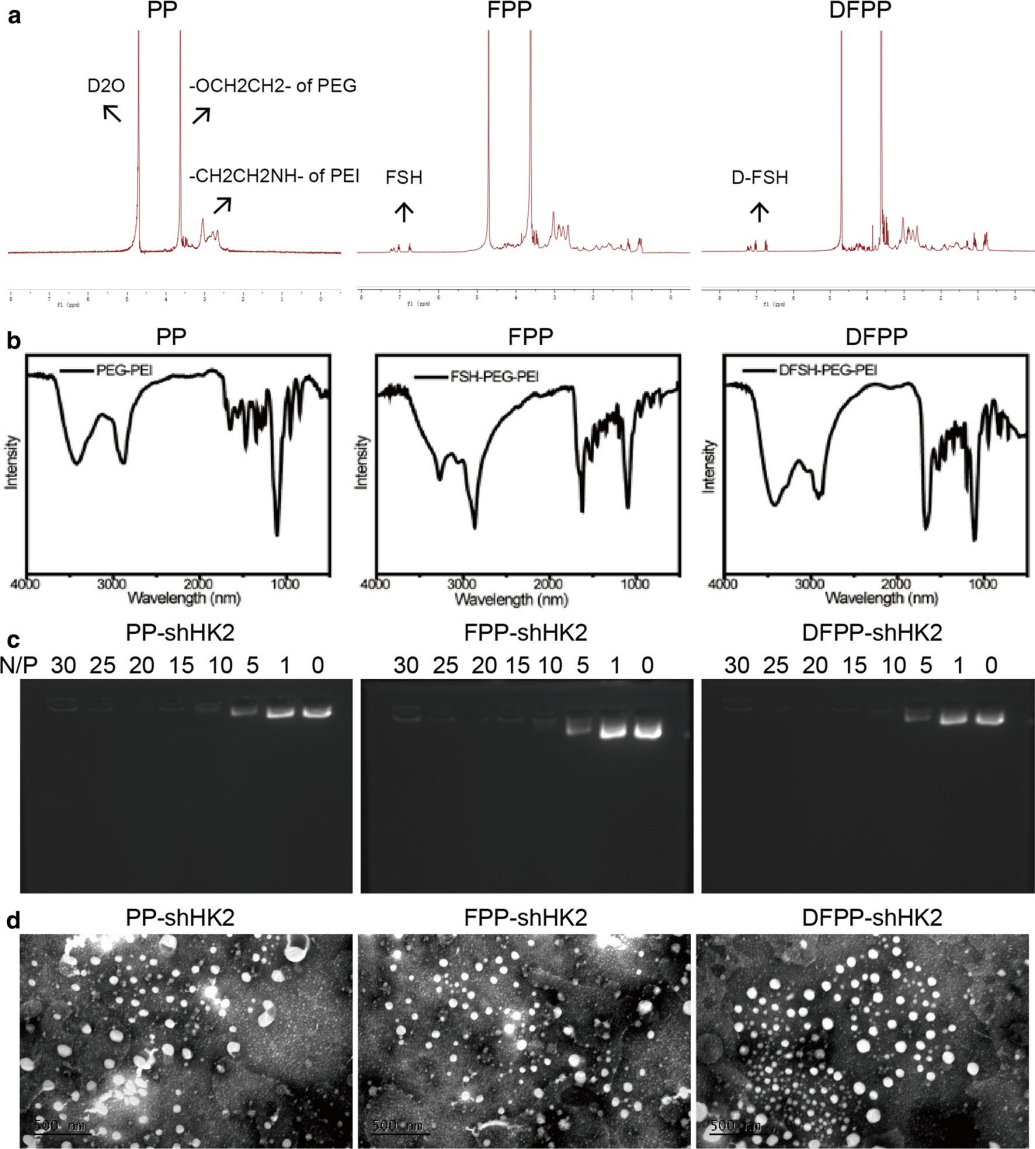
Characterization of nanoparticle complexes. **a** ^1^H-NMR spectroscopy of the copolymers PP, FPP and DFPP. The peaks at 4.6–4.7 ppm were chemical shifts due to D_2_O. PP had peaks at 3.5–3.6 ppm (from PEG) and 2.4–3.0 ppm (from PEI). FPP and DFPP had extra peaks at 7.0–7.2 ppm due to the FSH peptides. **b** FTIR of the copolymers PP, FPP and DFPP. **c** The agarose gel electrophoresis assays of PP-shHK2, FPP-shHK2 and DFPP-shHK2 at different N/P ratios. **d** Transmission electron microscopy images of PP-shHK2, FPP-shHK2 and DFPP-shHK2. Scale bar, 500 nm

FSH-derived peptides have been used for targeted therapy of ovarian cancer in terms of the limited expression of FSHR [24, 29]. The synthetic peptide of the FSH β chain sequence (amino acids 33–53) can bind FSHR by mimicking FSH [30]. We previously revealed the high selectivity of drug delivery mediated by FSH β 33–53 peptides [25]. However, natural L-amino acids are not stable enough in the bloodstream. Studies have shown that D-amino acids based on a retro-inverso design are resistant to endogenous proteolysis and have long-lasting effects [31, 32]. The retro-inverso FSH β 33–53 peptides exhibited similar advantages in our previous study [28]. To enhance the selectivity of PEG-PEI (PP) as an HK2 shRNA carrier, we synthesized FSH β 33–53 peptide-conjugated PEG-PEI (FPP) and retro-inverso FSH β 33–53 peptide-conjugated PEG-PEI (DFPP). The copolymers were characterized by ^1^H nuclear magnetic resonance (^1^H-NMR) and Fourier transform infrared spectrometer (FTIR). The spectra of FPP and DFPP simultaneously showed the peaks of the peptides, PEG and PEI (Fig. 2a, b, and Additional file 1: Figure S2), which indicated the successful synthesis of the copolymers.

Then, the copolymers and HK2 shRNA plasmids were combined via electrostatic interactions at different N/P (nitrogen to phosphorus) ratios and named PP-shHK2, FPP-shHK2 and DFPP-shHK2. Agarose gel electrophoresis was used to investigate the combination of plasmids and copolymer (Fig. 2c). The loading ability of the copolymers increased as the N/P ratio increased. When the N/P ratio was higher than or equal to 10, the plasmids could be completely loaded by the copolymers. Nanoparticle complexes with an N/P ratio of at least 25 had higher transfection efficiency and were more stable in our previous studies. Thus, an N/P ratio of 25 was used in subsequent experiments. The average particle DLS size of PP-shHK2 was 125.7 ± 42.35 nm, and the average particle DLS size of FPP-shHK2 and DFPP-shHK2 were 152.4 ± 20.15 nm and 163.4 ± 26.45 nm, respectively. The zeta potential values of PP-shHK2, FPP-shHK2 and DFPP-shHK2 were 42.30 ± 2.27 mV, − 26.70 ± 2.61 mV and − 29.40 ± 1.67 mV, respectively (Additional file 1: Table S1). The absolute value of zeta potential at N/P ratio of 25 was greater than those values at other N/P ratios, which indicated better stability. All of the complexes exhibited relatively homogeneous spherical shapes under transmission electron microscopy (Fig. 2d).

### Retro-inverso FSH β 33–53 peptide enhanced the gene knockdown effects of HK2 shRNA-loaded nanoparticles in vitro

**Fig. 3:**
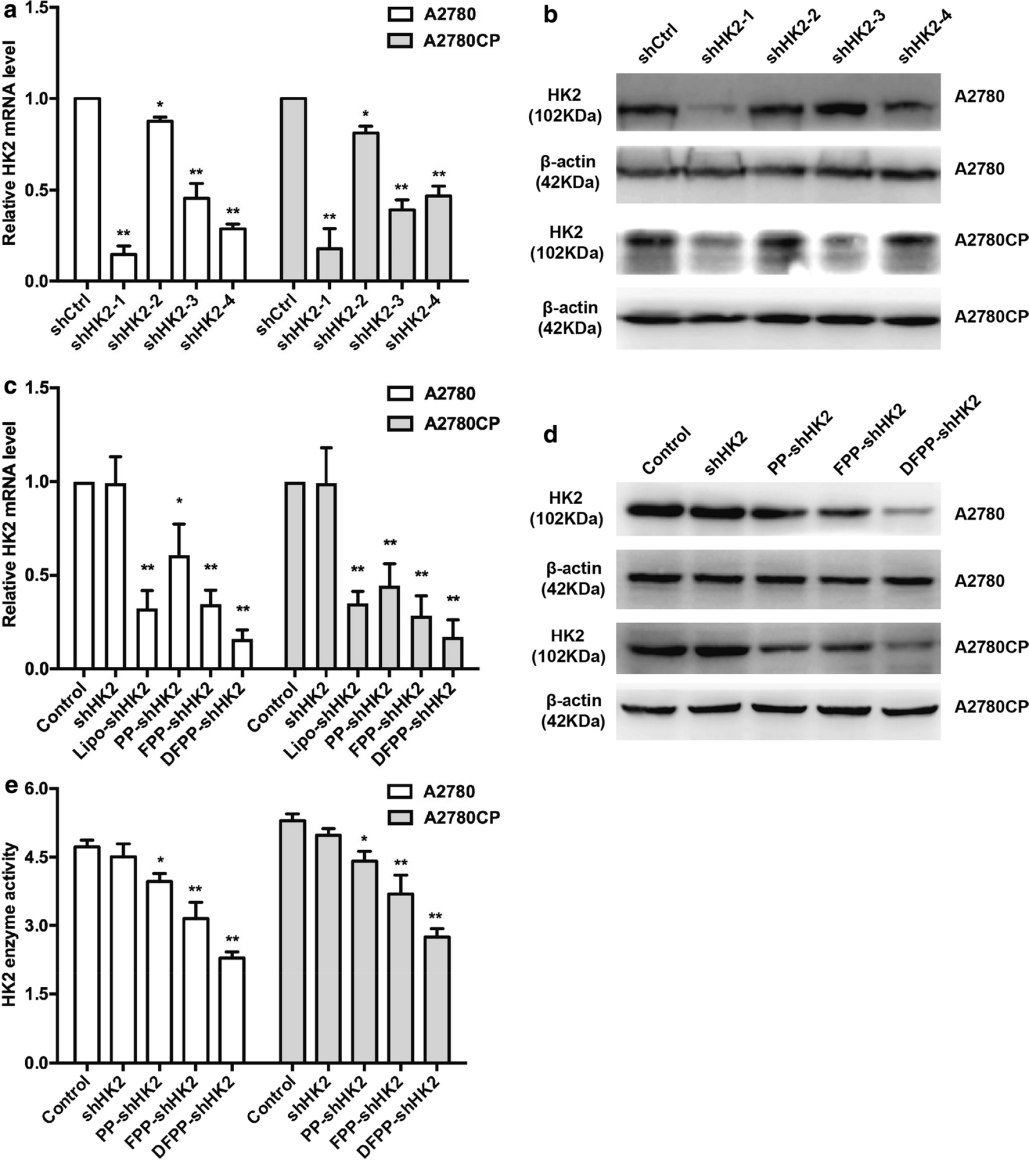
HK2 knockdown induced by HK2 shRNA-loaded nanoparticles in ovarian cancer cells. **a** Relative HK2 mRNA levels according to qRT-PCR and **b** HK2 protein levels according to western blotting in A2780 and A2780CP cells treated with different HK2 shRNAs for 24 h and 48 h, respectively. The concentration of the HK2 shRNA plasmid was 1.0 μg/ml. The transfection reagent was added to the culture medium. **c** Relative HK2 mRNA levels and **d** HK2 protein levels in A2780 and A2780CP cells treated with HK2 shRNA-loaded nanoparticles for 24 h and 48 h, respectively. The nanoparticles were diluted to a plasmid-equivalent concentration of 1.0 μg/ml. The transfection reagent was added in the lipo-shHK2 group but not added in other groups. **e** Cellular HK2 enzyme activity changes in A2780 and A2780CP cells treated with HK2 shRNA-loaded nanoparticles. *P < 0.05, **P < 0.01 vs. control

To screen for the most effective HK2 shRNA sequence, we detected the knockdown effects of four sequences in ovarian cancer cells by means of transfection reagents. As shown in Fig. 3a, b, shHK2-1 most effectively reduced the expression levels of HK2 mRNA and protein compared with the control treatment and the other three shRNA sequences in A2780 and A2780CP cells. Thus, the shHK2-1 plasmid was loaded into PEG-PEI copolymers (named PP-shHK2, FPP-shHK2 and DFPP-shHK2) and used in subsequent experiments.

Then, the knockdown effects of nanoparticle complexes on HK2 were confirmed in ovarian cancer cells (Fig. 3c, d). Both A2780 and A2780CP cells exhibited a decrease in HK2 levels after treatment with nanoparticle complexes containing HK2 shRNA (shHK2-1 sequence). Treatment with the naked shHK2 plasmid did not reduce HK2 expression when Lipofectamine 2000 was not added to the culture medium. Even PP-shHK2, which was without FSH peptide modification, could reduce the level of HK2 to some extent, which was consistent with the high transfection efficiency of PEI as a polymeric vector [33]. The knockdown effect of PP-shHK2 was weaker than the shHK2 plasmid in the presence of Lipofectamine 2000. The attachment of targeting ligands promotes tumor specificity and transfection efficiency and reduces the toxicity of PEI polymers [33, 34]. In this study, the FSH β 33–53 peptide and retro-inverso FSH β 33–53 peptide were used to target FSHR-positive ovarian cancer. Compared with PP-shHK2 and the transfected shHK2 plasmid, the HK2 shRNA-loaded nanoparticles with FSH peptide modification, FPP-shHK2 and DFPP-shHK2, greatly reduced HK2 expression. DFPP-shHK2 showed the greatest knockdown effect. Accordingly, cellular HK2 enzyme activity was significantly decreased in both A2780 and A2780CP cells after FPP-shHK2 and DFPP-shHK2 treatment (Fig. 3e), which could be due to the decreased levels of HK2 expression. These data indicated that HK2 shRNA-loaded nanoparticles with FSH peptides or retro-inverso FSH peptide modification effectively attenuated HK2 expression even without the help of transfection reagents, which further enhanced the feasibility of the in vivo administration of RNAi drugs and nanoparticles.

### HK2 knockdown by retro-inverso FSH β 33–53 peptide-conjugated nanoparticles facilitated a shift in the glucose metabolism pattern of ovarian cancer cells

In contrast to normal cells, cancer cells prefer aerobic glycolysis even at normal oxygen levels. One possible explanation is that cancer cells have an intense metabolic demand without efficient ATP production [35]. Cancer cells generate glycolytic intermediates that are available for the synthesis of amino acids, nucleotides, and fatty acids to meet the demands of fast proliferation. Another possible explanation is that the switch to aerobic glycolysis is linked to therapeutic resistance [36]. Highly invasive ovarian cancer cells present a more intensive glycolytic phenotype than less invasive ovarian cancer cells [37, 38]. HK2 is crucial for aerobic glycolysis and is overexpressed in ovarian cancer [15–17].

**Fig. 4:**
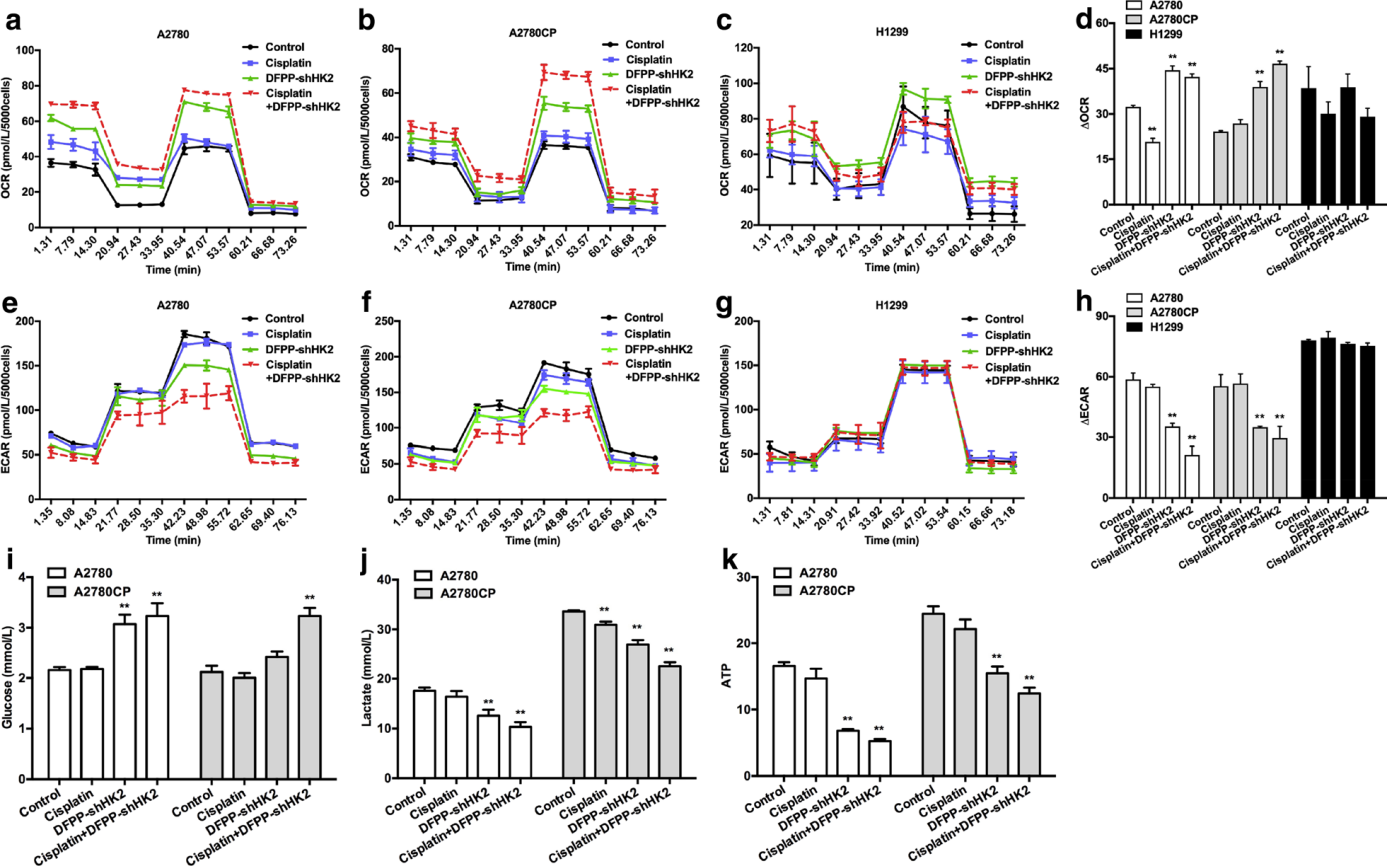
HK2 knockdown induced by DFPP-shHK2 facilitated a shift in the glucose metabolism pattern of ovarian cancer cells. A2780, A2780CP and H1299 cells were treated with DFPP-shHK2 and cisplatin alone or in combination for 24 h. The concentration of cisplatin was 2.5 μg/ml. DFPP-shHK2 was used at a plasmid-equivalent concentration of 1.0 μg/ml. The OCR was measured using the Seahorse XF96 analyzer in A2780 (**a**), A2780CP (**b**) and H1299 (**c**) cells. Oligomycin, FCCP, rotenone and antimycin A were added to challenge the cells. **d** The OCR changes showing FCCP-stimulated oxygen consumption compared to the baseline. The ECAR was measured in A2780 (**e**), A2780CP (**f**) and H1299 (**g**) cells challenged by glucose, oligomycin and 2-DG. **h** The ECAR changes showing oligomycin-stimulated glycolysis compared to the baseline. **i** Glucose level in medium. **j** Lactate level in medium. **k** Cellular ATP levels. *P < 0.05, **P < 0.01 vs. control

To determine whether knockdown of HK2 reverses the glucose metabolism pattern, OCR and ECAR were evaluated in cisplatin-sensitive and cisplatin-resistant ovarian cancer cells. A2780 and A2780CP cells treated with DFPP-shHK2 showed significantly enhanced FCCP-stimulated oxygen consumption, suggesting that HK2 silencing upregulated mitochondrial respiration (Fig. 4a–d). Oligomycin was used to inhibit ATP production by the electron transport chain and to evaluate the glycolytic reserves of cells. A2780 and A2780CP cells treated with DFPP-shHK2 exhibited a significant decrease in oligomycin-stimulated ECAR, indicating the reversal of the Warburg effect in cells (Fig. 4e–h). Compared to A2780 and A2780CP cells, no obvious shift in glucose metabolism pattern was observed in the H1299 cells with low expression of HK2 and FSHR. Glucose consumption, lactate production and cellular ATP levels were further measured to determine the glycolytic shift (Fig. 4i–k). After treatment with DFPP-shHK2 for 24 h, lactate levels in the medium and cellular ATP levels significantly decreased, while glucose levels in the medium were increased in both A2780 and A2780CP cells. The effects of cisplatin alone or in combination with DFPP-shHK2 on glucose metabolism were also investigated. As shown in Fig. 4, both A2780 and A2780CP cells exhibited a significant shift towards OXPHOS-dependent glucose metabolism after treatment with a combination of cisplatin and DFPP-shHK2 in comparison with that in cells treated with cisplatin alone. Taken together, these data suggested that knockdown of HK2 facilitated a shift towards normal oxidative glucose metabolism even in cisplatin-resistant ovarian cancer cells. The underlying mechanism could be associated with changes in mitochondrial function and biogenesis. After depletion of HK2, there was an increase in the activity of the electron transport chain complexes I/II/III/IV/V, peroxisome-proliferator-activated receptor-γ coactivator α (PGC1α) and mitochondrial transcription factor A (mTFA) [39].

### Antitumor effects of HK2 shRNA-loaded nanoparticles on chemosensitive ovarian cancer in vitro and in vivo

**Fig. 5:**
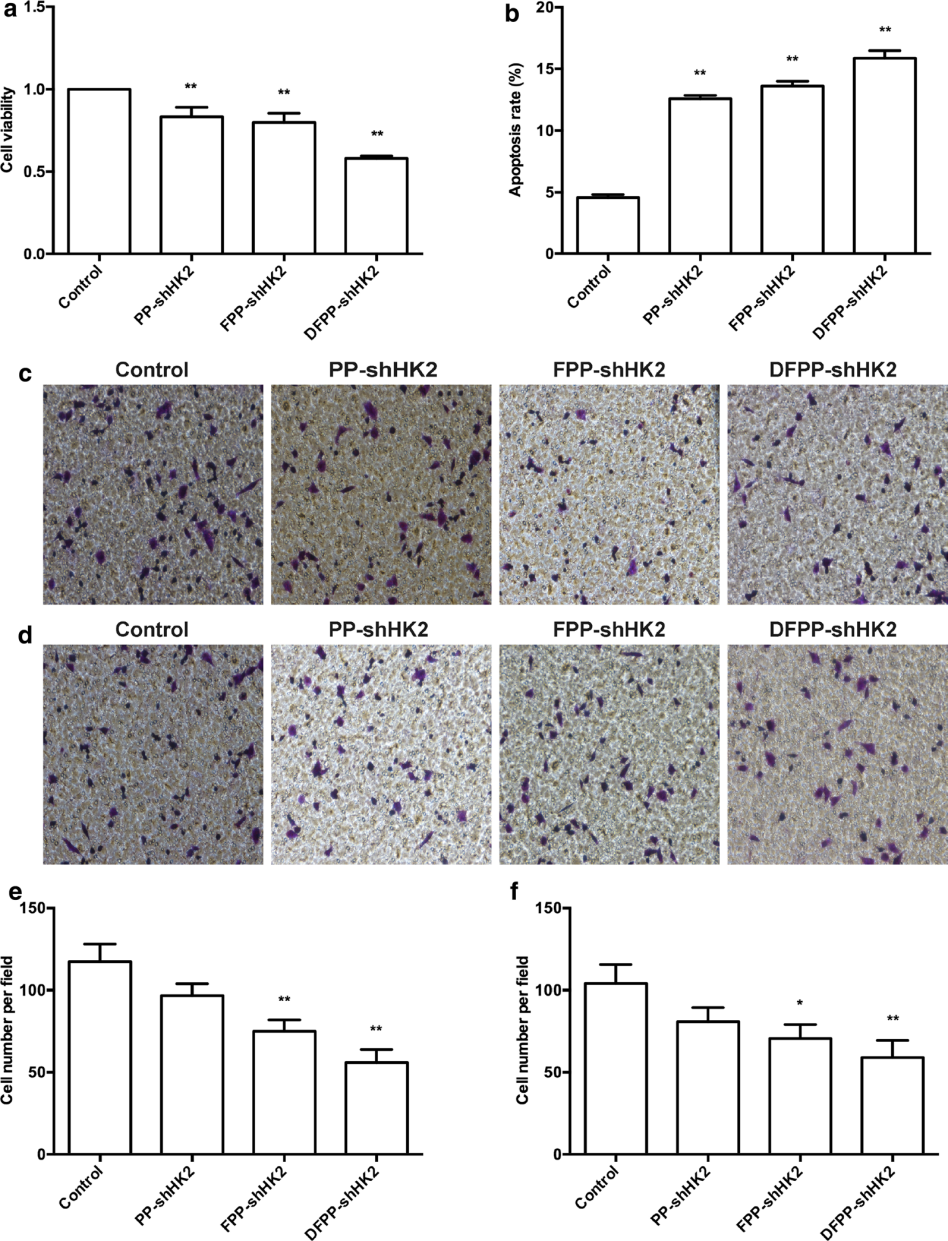
In vitro effects of HK2 shRNA-loaded nanoparticles on chemosensitive A2780 cells. A2780 cells were treated with HK2 shRNA-loaded nanoparticles at a plasmid-equivalent concentration of 1.0 μg/ml. **a** Cell viability according to a CCK-8 assay. **b** Cell apoptosis according to flow cytometry. Cell migration (**c**, **e**) and cell invasion (**d**, **f**) according to a Transwell assay. *P < 0.05, **P < 0.01 vs. control

To determine the effects of HK2 shRNA-loaded nanoparticles on the viability, migration, invasion and apoptosis of chemosensitive ovarian cancer cells, cisplatin-sensitive A2780 cells were used. As shown in Fig. 5, FPP-shHK2 and DFPP-shHK2 significantly inhibited the proliferation, invasion and migration and induced apoptosis of A2780 cells, especially DFPP-shHK2, which had the greatest effect on the knockdown of HK2 expression. In contrast, overexpression of HK2 could promote cell proliferation, migration, and invasion and the stemness of ovarian cancer cells via the FAK/ERK1/2 signaling pathway [17]. In addition, cell viability and apoptosis were also detected in cancer cells with low expression of HK2 and FSHR (Additional file 1: Figure S3). The anti-tumor effects of HK2 shRNA-loaded nanoparticles on OVCAR8 and H1299 cells were not as significant as on A2780 cells.

**Fig. 6:**
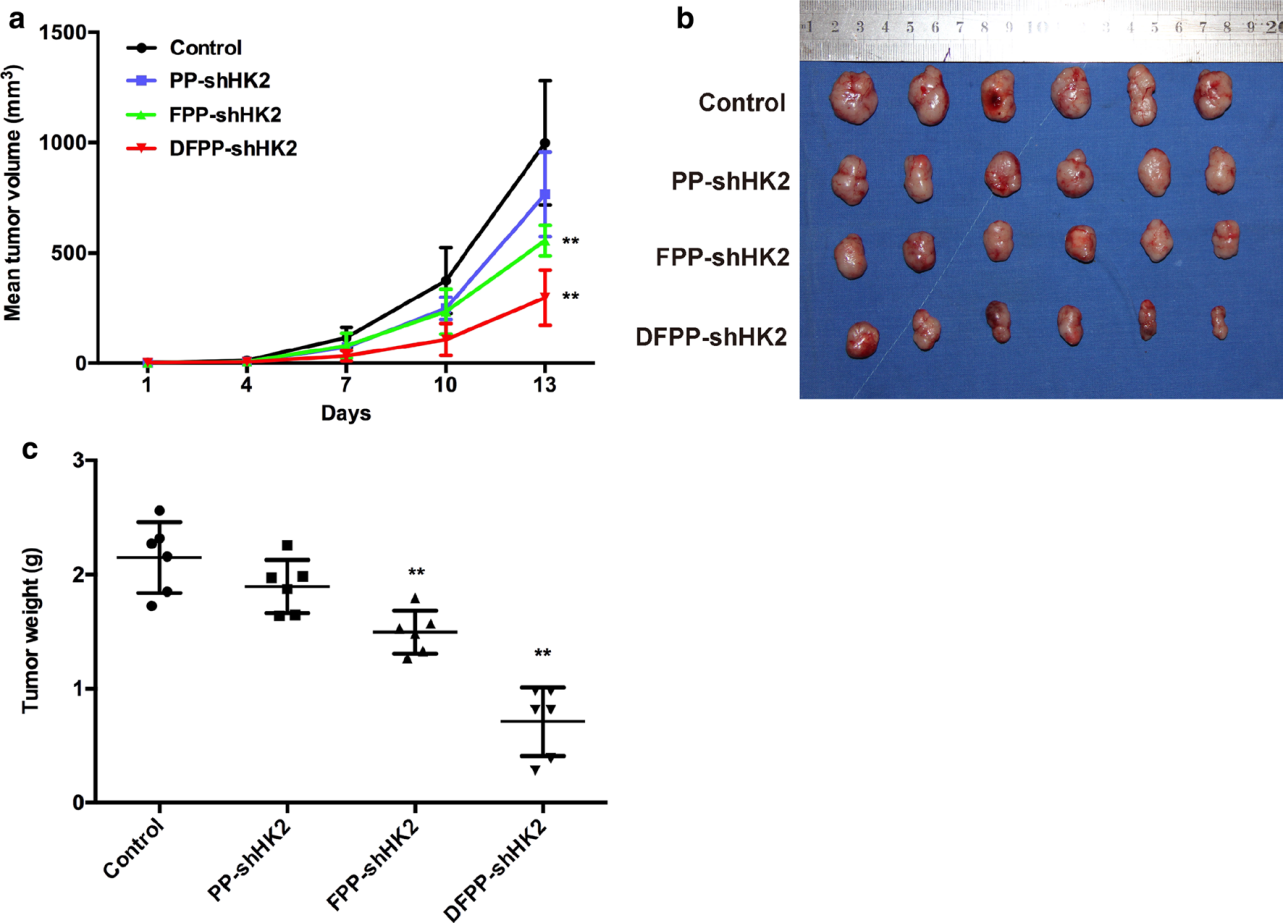
Antitumor effects of HK2 shRNA-loaded nanoparticles on chemosensitive A2780 tumor xenografts. The nude mice bearing cisplatin-sensitive A2780 tumor xenografts received an intravenous administration of saline, PP-shHK2, FPP-shHK2 or DFPP-shHK2 on days 1, 4, 7 and 10. **a** Tumor volume changes. **b** Tumor photos. **c** Tumor weights at the study end point. *P < 0.05, **P < 0.01 vs. control

The antitumor effects of HK2 shRNA-loaded nanoparticles were further investigated using nude mice bearing cisplatin-sensitive A2780 tumor xenografts. PP-shHK2, FPP-shHK2 or DFPP-shHK2 nanoparticles were intravenously administered. As shown in Fig. 6, FPP-shHK2 and DFPP-shHK2 significantly suppressed tumor growth compared with the control treatment. DFPP-shHK2 treatment exhibited the highest antitumor effect, with an inhibition rate of 70.1%. There was no difference between the PP-shHK2 and control groups, which might be due to the weak knockdown effect of PP-shHK2 on HK2 expression.

These data were consistent with the data from the in vitro experiments and further indicated that FPP-shHK2 and DFPP-shHK2 effectively inhibited tumor growth in cisplatin-sensitive ovarian cancer. The possible reasons for this could involve the increased accumulation of HK2 shRNA in the tumor sites, which was mediated by FSH peptides, and the subsequent suppression of the glycolysis of cancer cells. Although the related downstream molecular mechanisms remain unclear, the effects of metabolites on tumors have been partially elucidated. Lactate has been identified as a primary fuel for the TCA cycle, and lung cancer cells prefer to use lactate as an energy source for tumor metabolism [40, 41]. It has been indicated that the purpose of the Warburg effect is to generate lactate for carcinogenesis [42]. Lactate is involved in the development of cancer, including angiogenesis, immune escape, and cell migration [43]. The enhancement by lactate of the migratory potential of cancer cells occurs in a dose-dependent manner [44]. Here, knockdown of HK2 greatly reduced extracellular lactate levels, which could contribute to the antitumor effects in vitro and in vivo.

### HK2 knockdown by retro-inverso FSH β 33–53 peptide-conjugated nanoparticles improved cisplatin sensitivity in vitro

**Fig. 7:**
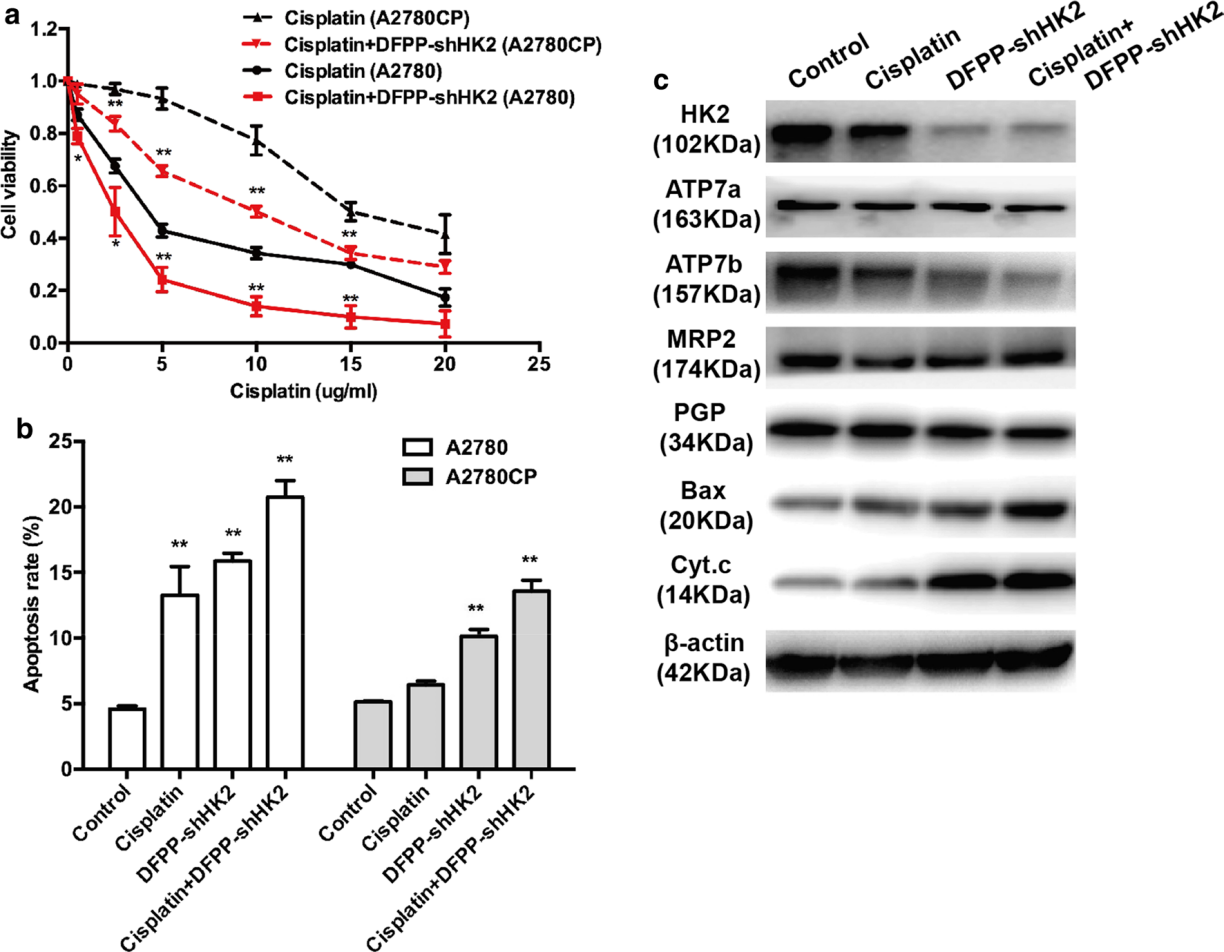
HK2 knockdown induced by DFPP-shHK2 improved the cisplatin sensitivity of ovarian cancer cells. **a** Cell viability in A2780 and A2780CP cells treated with DFPP-shHK2 combined with cisplatin. The concentration of cisplatin ranged from 0.5 μg/ml to 20 μg/ml. DFPP-shHK2 was used at a plasmid-equivalent concentration of 1.0 μg/ml. **b** Cell apoptosis according to flow cytometry in A2780 and A2780CP cells treated with DFPP-shHK2 and cisplatin alone or in combination. The concentration of cisplatin was 2.5 μg/ml. **c** The expression levels of cisplatin resistance-associated proteins according to western blotting in A2780CP cells treated with DFPP-shHK2 and cisplatin alone or in combination for 48 h. *P < 0.05, **P < 0.01 vs. control

An increasing number of studies have revealed that the enhancement of aerobic glycolysis contributes to chemoresistance [6, 45, 46]. An increase in glycolysis has been observed in chemoresistant cancer cells, and increased intracellular ATP levels can induce a drug-resistant phenotype [47]. Signaling pathways, such as the HIF-1α signaling pathway, that are activated by dysregulated metabolism may also contribute to chemoresistance. HK2 overexpression is related to the chemoresistance of glioblastoma, acute myeloid leukemia, colorectal cancer and ovarian cancer [15, 48–50]. To further investigate the effect of HK2 knockdown on ovarian cancer chemoresistance, cisplatin-resistant A2780CP cells were treated with DFPP-shHK2 combined with cisplatin. Both A2780 and A2780CP cells showed an increased sensitivity to cisplatin when treated with the DFPP-shHK2 combination (Fig. 7a, b). Proliferation was inhibited, and apoptosis was enhanced even in cisplatin-resistant A2780CP cells. The 24-h half maximal inhibitory concentrations (IC_50_) of cisplatin for the A2780CP and DFPP-shHK2-treated A2780CP cells were 16.5 and 10 μg/mL, respectively. The inhibitory effects could be linked to the shift of glycolysis to OXPHOS-dependent glucose metabolism after depletion of HK2. The reduced levels of glycolytic intermediates such as lactate and reduced ATP levels could not sustain the demands of ovarian cancer cells to resist the killing power of cisplatin.

Furthermore, we investigated the changes in cisplatin resistance-related proteins after HK2 shRNA-loaded nanoparticle treatment. The expression level of ATP7b, which is an ATP-related transport protein that promotes cisplatin resistance by pumping cisplatin out of cancer cells, was significantly decreased by DFPP-shHK2-induced HK2 knockdown (Fig. 7c). However, other cisplatin transport proteins, such as ATP7a, PGP and MRP2, showed no changes. The expression levels of the mitochondrial apoptosis-associated proteins Bax and cytochrome c were increased after DFPP-shHK2 treatment. These data suggested that HK2 knockdown induced by DFPP-shHK2 effectively improved the cisplatin sensitivity of ovarian cancer cells by regulating cisplatin transport proteins and increasing apoptosis through the mitochondrial pathway.

### Antitumor effects of retro-inverso FSH β 33–53 peptide-conjugated nanoparticles on cisplatin-resistant tumor xenografts

**Fig. 8:**
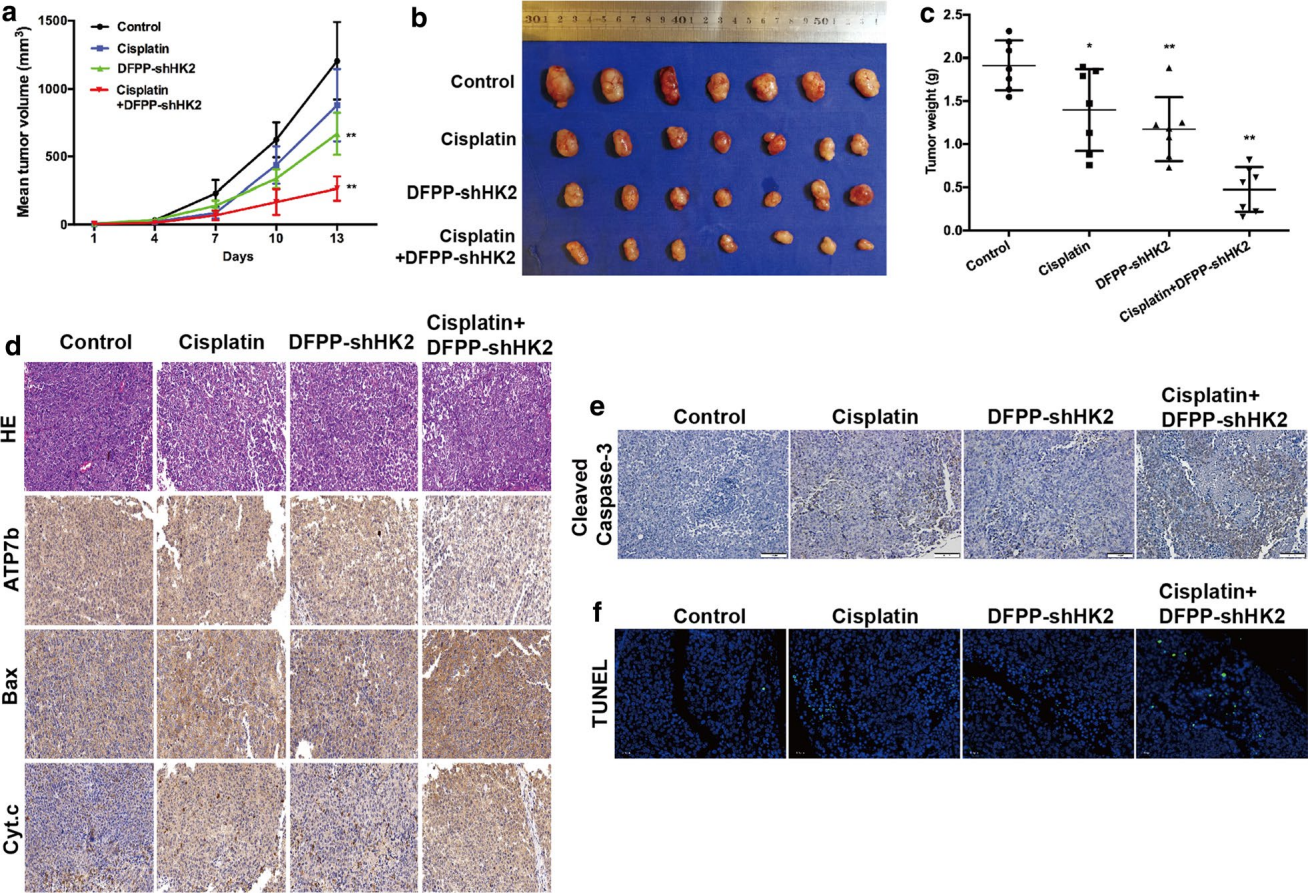
Antitumor effects of DFPP-shHK2 on cisplatin-resistant A2780CP tumor xenografts. Nude mice bearing cisplatin-resistant A2780CP tumor xenografts received an intravenous administration of DFPP-shHK2 and cisplatin alone or in combination on days 1, 4, 7 and 10. **a** Tumor volume changes. **b** Tumor photos. **c** Tumor weights at the study end point. *P < 0.05, **P < 0.01 vs. control. **d** The expression of cisplatin resistance-associated proteins in A2780CP tumor xenografts according to immunohistochemistry (200X). **e** IHC staining of cleaved caspase-3 in A2780CP tumor xenografts (200X). **f** TUNEL staining in A2780CP tumor xenografts (200X)

To evaluate the in vivo effects of HK2 depletion, nude mice bearing cisplatin-resistant A2780CP tumor xenografts were intravenously administered DFPP-shHK2 and cisplatin alone or in combination. As shown in Fig. 8a–c, DFPP-shHK2 significantly suppressed tumor growth, while the inhibitory effect in cisplatin-resistant A2780CP tumors was obviously lower than that in cisplatin-sensitive A2780 tumors. When the mice were treated with DFPP-shHK2 in combination with cisplatin, both the tumor volumes and tumor weights were dramatically reduced, with an inhibition rate of 78.1%. Interestingly, the expression level of the cisplatin resistance-associated protein ATP7b in A2780CP tumor xenografts showed no changes in the cisplatin alone group but showed a decrease in the DFPP-shHK2 combination group. Bax and cytochrome c expression were increased in the cisplatin alone and combination groups compared with those in the control group (Fig. 8d). The cleaved caspase-3 expression (Fig. 8e) and TUNEL assay (Fig. 8f) indicated that apoptotic tumor cells were increased after DFPP-shHK2 treatment. Together, the results show that targeting HK2 could be an effective therapeutic strategy for cisplatin-resistant ovarian cancer, although the molecular mechanisms of drug resistance that are affected by aerobic glycolysis need further elucidation.

### In vivo toxicity of HK2 shRNA-loaded nanoparticles

**Fig. 9:**
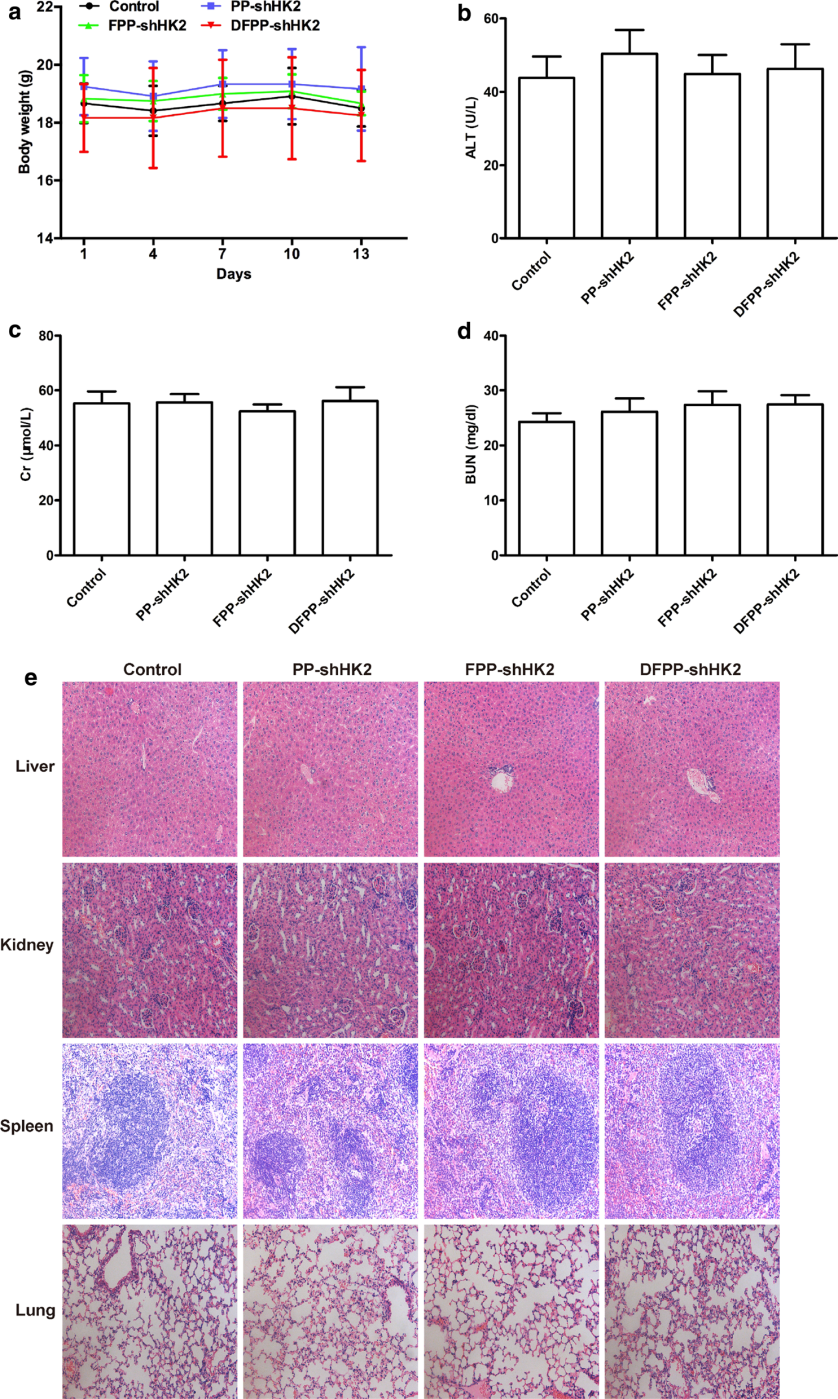
In vivo toxicity of HK2 shRNA-loaded nanoparticles. Nude mice bearing cisplatin-sensitive A2780 tumor xenografts received an intravenous administration of saline, PP-shHK2, FPP-shHK2 or DFPP-shHK2 on days 1, 4, 7 and 10. **a** Body weight changes of the mice. The blood levels of ALT (**b**), Cr (**c**) and BUN (**d**) in all mice. **e** HE staining of the liver, kidney, spleen and lung (200X)

The systemic toxicity of small molecular inhibitors of HK2 has limited their clinical use. High levels of HK2 inhibitors that sufficiently suppress tumor growth might simultaneously lead to toxicity in normal cells. Additionally, the polymers used in the delivery system easily accumulate in lung and liver tissues, which may cause toxic effects. It has been proven that the modification of targeting peptides on polymers helps to achieve tumor-specific delivery and overcome natural accumulation [34]. Since FSHR is overexpressed in different histologic types of ovarian cancer but not in non-ovarian healthy tissues [24], we designed FSHR-mediated nanocarriers for HK2 shRNA to increase tumor uptake and decrease toxicity by using the binding peptides of FSHR. As shown in Fig. 9, the in vivo toxicity of HK2 shRNA-loaded nanoparticles was evaluated in nude mice bearing A2780 tumor xenografts. The body weights were not significantly reduced in mice treated with HK2 shRNA-loaded nanoparticles during the study period. The blood levels of ALT, Cr and BUN exhibited no significant differences among all groups. HE staining of the liver, kidney, spleen and lung did not show any obvious damage.

Although the relationship between FSH, FSHR and ovarian cancer remains controversial, the safety and effectiveness of FSHR- and FSH-derived peptides has been examined in studies of the targeted immunotherapy of ovarian cancer. Targeting chimeric receptors for T cell activation using full-length FSH produces strong therapeutic effects against ovarian cancer in vivo without any measurable toxicity, even in the presence of tumor-free ovaries [24]. Similar results have been observed in a study of FSHR-redirected T-cells using FSH fragments, including the FSH β 33–53 peptide [29].

### Conclusions

In this study, we developed HK2 shRNA-loaded nanoparticles with FSH β 33–53 or retro-inverso FSH β 33–53 peptide modification to target tumor metabolism and tumor growth in ovarian cancer. These nanoparticle complexes effectively suppressed HK2 expression, resulting in a reversal of glycolytic-based glucose metabolism and strong antitumor effects even in cisplatin-resistant ovarian cancer with negligible systemic toxicity. These features make it a potential therapeutic alternative for the treatment of ovarian cancer. Further studies will be needed to examine the biodistribution of drugs delivered by FSHR-mediated nanocarriers.

## Methods

### Cell lines

A2780 and A2780CP human ovarian cancer cells (A2780CP cells are the cisplatin-resistant counterpart of A2780) were obtained from Professor Yu Yinhua (MD Anderson Cancer Center, USA). Human ovarian cancer cell line OVCAR8, lung cancer cell line H1299 and liver cancer cell line HepG2 were archived in our laboratory. The cells were grown in RPMI-1640 medium supplemented with 10% fetal bovine serum. All the cell lines were cultured at 37 °C in an incubator with 5% CO_2_.

## Materials

The FSH β 33–53 peptide (YTRDLVYKDPARPKIQKTCTF) and retro-inverso FSH β 33–53 peptide (FTCTKQIKPRAPDKYVLDRTY) were synthesized by ChinaPeptides (China). PEI (polyethylenimine, branched, MW 25,000 Da) was purchased from Sigma-Aldrich (USA). PEG (MAL-PEG3500-SCM, maleimide PEG succinimidyl carboxymethyl ester) was purchased from JenKem Technology (China). The HK2 shRNA was synthesized by Genechem (China). The sequences were as follows: shHK2-1, 5′-CTGTGAAGTTGGCCTCATT-3′; shHK2-2, 5′-TGAGTTTGACCAGGAGATT-3′; shHK2-3, 5′-GTAACATTCTCATCGATTT-3′; shHK2-4, 5′-CTGGCTAACTTCATGGATA-3′. The vector GV248, hU6-MCS-Ubiquitin-EGFP-IRES-puromycin, was used for the plasmids construction.

### Preparation and characterization of nanoparticles

A mixture of FSH β 33–53 peptide or retro-inverso FSH β 33–53 peptide with PEG was magnetically stirred for 6 h and then ultrafiltered using an Amicon Ultra-4 mL Filter Unit (Millipore, USA). PEI was dissolved in distilled water and adjusted to pH 7.0 by adding HCl. Next, PEG with or without polypeptides was mixed with PEI, and the mixture was magnetically stirred overnight and then ultrafiltered.

The conjugation of FSH β 33–53 peptide, PEG and PEI was verified using ^1^H-NMR spectroscopy (AVANCE III HD 400MHz, Bruker BioSpin International, Switzerland) and FTIR (Nicolet 6700, Thermofisher, USA). The conjugation of plasmids and copolymer was detected by agarose gel electrophoresis. The particle size, zeta potential and morphology were detected using a transmission electron microscope (JEOL Ltd, Japan) and Malvern Zetasizer Autosizer 4700 (Malvern Instruments, Ltd., Malvern, UK).

### CCK-8 assay

The cytotoxicities of the nanoparticles and cisplatin were assessed by a cell counting kit-8 (CCK-8) assay (Dojindo, Japan). A total of 3.5 × 10^3^ cells per well were seeded into 96-well plates overnight and incubated with nanoparticles or cisplatin at different concentrations for 24 h.

### Cell apoptosis

After treatment with nanoparticles or cisplatin for 24 h, the cells were collected, washed twice with cold PBS, resuspended in binding buffer and stained with FITC-annexin V/PI. The samples were detected using a FACS Calibur flow cytometer (BD Biosciences, USA). Data were analyzed with FlowJo software (USA).

### Cell invasion and migration

For the cell migration assay, after treatment with nanoparticles or cisplatin for 24 h, the A2780 cells were collected, resuspended in serum-free medium and added into the upper Transwell chambers. RPMI 1640 medium containing 10% FBS was added to the lower chambers. After incubation for 24 h, the migrating cells on the lower surface were fixed, stained and counted. Cell invasion assays were conducted in a similar manner using Matrigel-coated transwell chambers.

### Quantitative real-time PCR

Total RNA was extracted and reverse-transcribed into cDNA. Quantitative real-time PCR (qRT-PCR) was conducted using PrimeScript RT Master Mix (Takara, Japan) according to the manufacturer’s instructions. All PCRs were performed in triplicate. The sequences of primers were as follows: (1) GAPDH, 5′-GGGAAGGTGAAGGTCGGAGT-3′ and 5′-GGGGTCATTGATGGCAACA-3′; (2) HK2, 5′-GCATCATAACCATTCCCATT-3′ and 5′-TTAGTGTCCCCATCCTGTAG-3'.

### Western blotting

Protein lysates were obtained using RIPA buffer supplemented with protease inhibitor. The protein extracts were separated by SDS-PAGE and transferred to PVDF membranes, which were incubated with the following primary antibodies: FSHR (Abcam), HK2 (Abcam), ATP7b (Abcam), PGP (Abcam), Bax (Abcam), cleaved caspase 3 (Abcam), cytochrome C (Cyt.c) (Abcam), GAPDH (CST) and β-actin (CST). Then, the membranes were incubated with HRP-conjugated secondary antibody (Abcam). The bands were visualized by chemiluminescence using the ImageQuant LAS4000 system (GE Healthcare LifeSciences).

### Metabolic analysis

Lactate production was measured using a lactate assay kit (BioVision, USA). Glucose consumption was assessed with a glucose assay kit (Sigma-Aldrich). After treatment with nanoparticles for 24 h, A2780 or A2780CP cells were cultured in RPMI 1640 without phenol red for 24 h. The culture medium was collected to measure the lactate and glucose levels. After treatment with nanoparticles for 24 h, cellular hexokinase activity was measured using a hexokinase assay kit (Abcam). The ATP levels were detected with an ATP bioluminescence assay kit (Beyotime Technology, China).

### Seahorse metabolic assays

The extracellular acidification rates (ECARs) and oxygen consumption rates (OCRs) of the cells were detected with a Seahorse Bioscience XF96 Extracellular Flux Analyzer (USA) by following the manufacturer’s protocols. The nanoparticle-pretreated cells were seeded in XF96 cell culture microplates overnight before the experiment. The glycolysis stress test was performed by adding glucose, oligomycin and 2-deoxyglucose (2-DG), and the mitochondrial stress test was performed by adding oligomycin, carbonylcyanide-p-trifluoromethoxyphenylhydrazone (FCCP), rotenone and antimycin A. The data were analyzed with the Seahorse XF96 analyzer (Agilent Technologies, USA).

### Tumor model

To evaluate the antitumor efficacy of the nanoparticles, A2780 xenografts were established. A total of 1 × 10^7^ A2780 cells were injected subcutaneously into the flanks of female Balb/c nude mice (Shanghai SLAC Laboratory Animal Center, China). When the tumors became palpable, the mice were randomly divided into four groups and administered HK2 shRNA-loaded nanoparticles and saline via the tail vein. The dosage of the nanoparticle complex was 5 mg/kg body weight. To evaluate the antitumor efficacy of the nanoparticles combined with cisplatin, A2780CP xenografts were established in a similar manner. The tumor-bearing mice were grouped and treated with retro-inverso FSH β 33–53 peptide-conjugated nanoparticles and cisplatin alone or in combination. The dosage of the nanoparticle complex was 5 mg/kg body weight, and the dosage of cisplatin was 3 mg/kg body weight.

During the study, the injections were performed on days 1, 4, 7 and 10. The body weight and tumor size were measured, and the tumor volume was calculated as π × larger diameter × (smaller diameter)^2^/6. The tumors and important organs were collected and fixed with 4% paraformaldehyde for histological analysis. Blood draws were performed to assess alanine transaminase (ALT), creatinine (Cr), and blood urea nitrogen (BUN). All animal experiments were conducted according to the principles for laboratory animal use and care and with approval from the Institutional Animal Care and Use Committee.

### Immunohistochemistry and TUNEL staining

Tumor tissues were fixed in paraformaldehyde and embedded in paraffin. For immunohistochemistry, tissue sections were deparaffinized and incubated with anti-FSHR (Abcam), anti-HK2 (Abcam), ATP7b (Abcam), Bax (Abcam), cytochrome C (Abcam) and cleaved caspase-3 (CST) antibodies. Then, the tissue sections were washed and incubated with HRP-conjugated secondary antibody. Diaminobenzidine and hematoxylin were used to stain tissues. The In Situ Cell Death Detection Kit (Roche, Switzerland) was used for TUNEL staining by following the manufacturer’s protocols.

### Statistical analyses

Student's t test and one-way ANOVA were used to determine the significant differences. Statistical analysis was performed using GraphPad Prism 6.0 (La Jolla, CA, USA) and SPSS 16.0 (SPSS Inc., USA).

## Supplementary information

**Additional file 1: Table S1.** DLS size and zeta potential of nanoparticles with different N/P ratios. **Figure S1.** Relative FSHR mRNA expression in human normal tissues (https://gtexportal.org/home/gene/FSHR). **Figure S2.** FTIR of FSH (A), DFSH (B), PEG (C), PEI (D), FSH-PEG (E) and DFSH-PEG (F). **Figure S3.** In vitro effects of HK2 shRNA-loaded nanoparticles on different cancer cell lines. (A) Cell viability according to a CCK-8 assay. (B) Cell apoptosis according to flow cytometry. **Figure S4.** In vitro effects of scramble shRNA-loaded nanoparticles on A2780 and A2780CP cells. A2780 and A2780CP cells were treated with scramble shRNA-loaded nanoparticles at a plasmid-equivalent concentration of 1.0 μg/ml. (A) Cell viability according to a CCK-8 assay. (B) Cell apoptosis according to flow cytometry.

## Data Availability

All data generated or analyzed during this study are included in this published article.

## Abbreviations

2-DG: 2-Deoxyglucose
ALT: Glutamic-pyruvic transaminase
BUN: Blood urea nitrogen
Cr: Creatinine
DFPP: Retro-inverso FSH β 33–53 peptide-conjugated PEG-PEI
ECARs: Extracellular acidification rates
EGFR: Epidermal growth factor receptor
FCCP: Trifluoromethoxy carbonylcyanide phenylhydrazone
FPP: FSH β 33–53 peptide-conjugated PEG-PEI
FSH: Follicle-stimulating hormone
FSHR: Follicle-stimulating hormone receptor0
G6P: Glucose-6-phosphate
HKs: Hexokinases
HK2: Hexokinase-2
OCARs: Oxygen consumption rates
OXPHOS: Oxidative phosphorylation
PEG: Polyethylene glycol
PEI: Polyethylenimine
PP: PEG-PEI; RNAi: RNA interference
shRNA: Short hairpin RNA
