# Supplementary material for: Enhanced antitumor effects of follicle-stimulating hormone receptor-mediated hexokinase-2 depletion on ovarian cancer mediated by a shift in glucose metabolism
Source: J Nanobiotechnology. 2020 Nov 7;18:161. doi: 10.1186/s12951-020-00720-4 (PMC7648390; doi:10.1186/s12951-020-00720-4)
Supplement: Supplementary file 1 — Additional file 1: Table S1. DLS size and zeta potential of nanoparticles with different N/P ratios. Figure S1. Relative FSHR mRNA expression in human normal tissues (https://gtexportal.org/home/gene/FSHR). Figure S2. FTIR of FSH (A), DFSH (B), PEG (C), PEI (D), FSH-PEG (E) and DFSH-PEG (F). Figure S3. In vitro effects of HK2 shRNA-loaded nanoparticles on different cancer cell lines. (A) Cell viability according to a CCK-8 assay. (B) Cell apoptosis according to flow cytometry. Figure S4. In vitro effects of scramble shRNA-loaded nanoparticles on A2780 and A2780CP cells. A2780 and A2780CP cells were treated with scramble shRNA-loaded nanoparticles at a plasmid-equivalent concentration of 1.0 μg/ml. (A) Cell viability according to a CCK-8 assay. (B) Cell apoptosis according to flow cytometry. [file 12951_2020_720_MOESM1_ESM.docx]

**Enhanced Antitumor Effects of Follicle-stimulating Hormone Receptor-mediated Hexokinase-2 Depletion on Ovarian Cancer Mediated by a Shift in Glucose Metabolism**

Meng Zhang^1†^, Qiyu Liu^1†^, Mingxing Zhang^1^, Cong Cao^2^, Xiaoxia Liu^1^, Mengyu Zhang^1^, Guiling Li^1^, Congjian Xu^1,3*^ and Xiaoyan Zhang^1*^

^1^ Obstetrics and Gynecology Hospital, Fudan University, Shanghai 200011, China

^2^ School of Materials Science & Engineering, Zhejiang Sci-Tech University, Hangzhou 310018, China

^3^ Shanghai Key Laboratory of Female Reproductive Endocrine Related Diseases, Shanghai 200011, China

^†^ Meng Zhang and Qiyu Liu equally contributed to this work

^*^ Correspondence: zhxy@fudan.edu.cn; [xucongjian@fudan.edu.cn](mailto:xucongjian@fudan.edu.cn)

**Supplementary Information**

**Table S1. DLS size and Zeta Potential of Nanoparticles with Different N/P Ratios**

|  | **N/P ratio** | **DLS size(nm)** | **PDI value** | **zeta potential**  **(mV)** |
| --- | --- | --- | --- | --- |
| PEG-PEI -shHK2 | 10 | 326.7±41.05 | 0.411±0.047 | 31.4±1.67 |
|  | 15 | 265.4±32.51 | 0.401±0.035 | 33.2±2.32 |
|  | 20 | 167.1±39.88 | 0.426±0.082 | 32.5±3.75 |
|  | 25 | 125.7±42.35 | 0.423±0.049 | 42.3±2.27 |
|  | 30 | 116.8±5.56 | 0.395±0.097 | 41.2±2.65 |
| FSH-PEG-PEI -shHK2 | 10 | 379.7±63.99 | 0.540±0.065 | -17.0±1.33 |
|  | 15 | 293.3±12.77 | 0.409±0.010 | -17.0±1.33 |
|  | 20 | 237.1±11.43 | 0.389±0.074 | -24.5±2.84 |
|  | 25 | 152.4±20.15 | 0.489±0.013 | -26.7±2.61 |
|  | 30 | 139.5±39.45 | 0.465±0.084 | -19.5±1.36 |
| D-FSH-PEG-PEI -shHK2 | 10 | 392.1±46.58 | 0.496±0.078 | -19.6±1.65 |
|  | 15 | 318.5±28.56 | 0.356±0.023 | -21.7±1.84 |
|  | 20 | 250.3±30.21 | 0.373±0.046 | -22.1±1.97 |
|  | 25 | 163.4±26.45 | 0.324±0.037 | -29.4±1.67 |
|  | 30 | 142.5±46.58 | 0.381±0.094 | -20.5±2.25 |


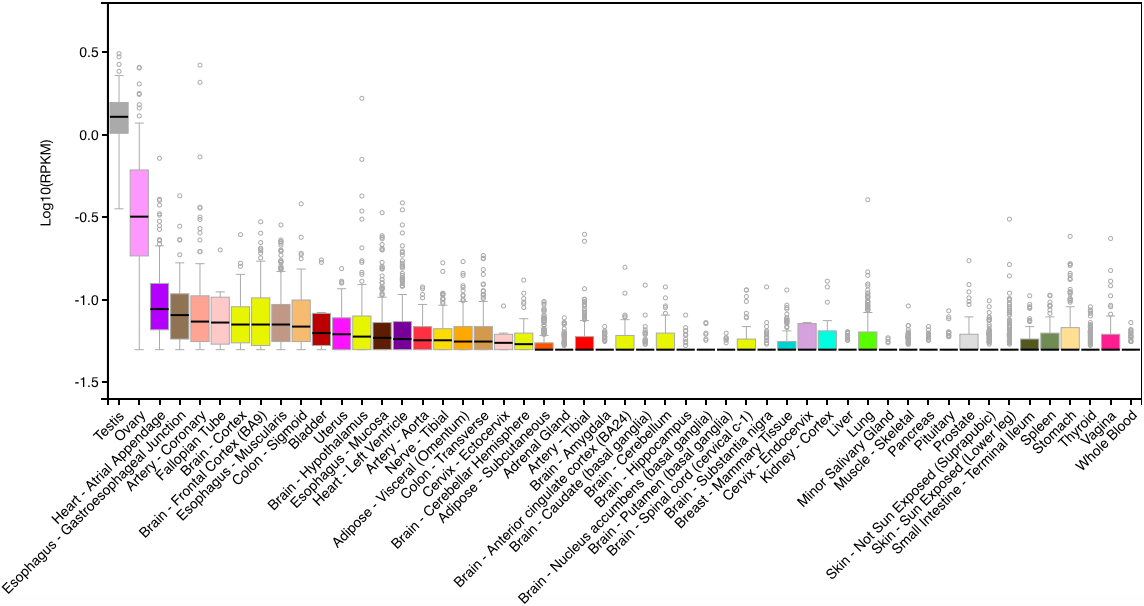


**Figure S1. Relative FSHR mRNA expression in human normal tissues.** (<https://gtexportal.org/home/gene/FSHR>)


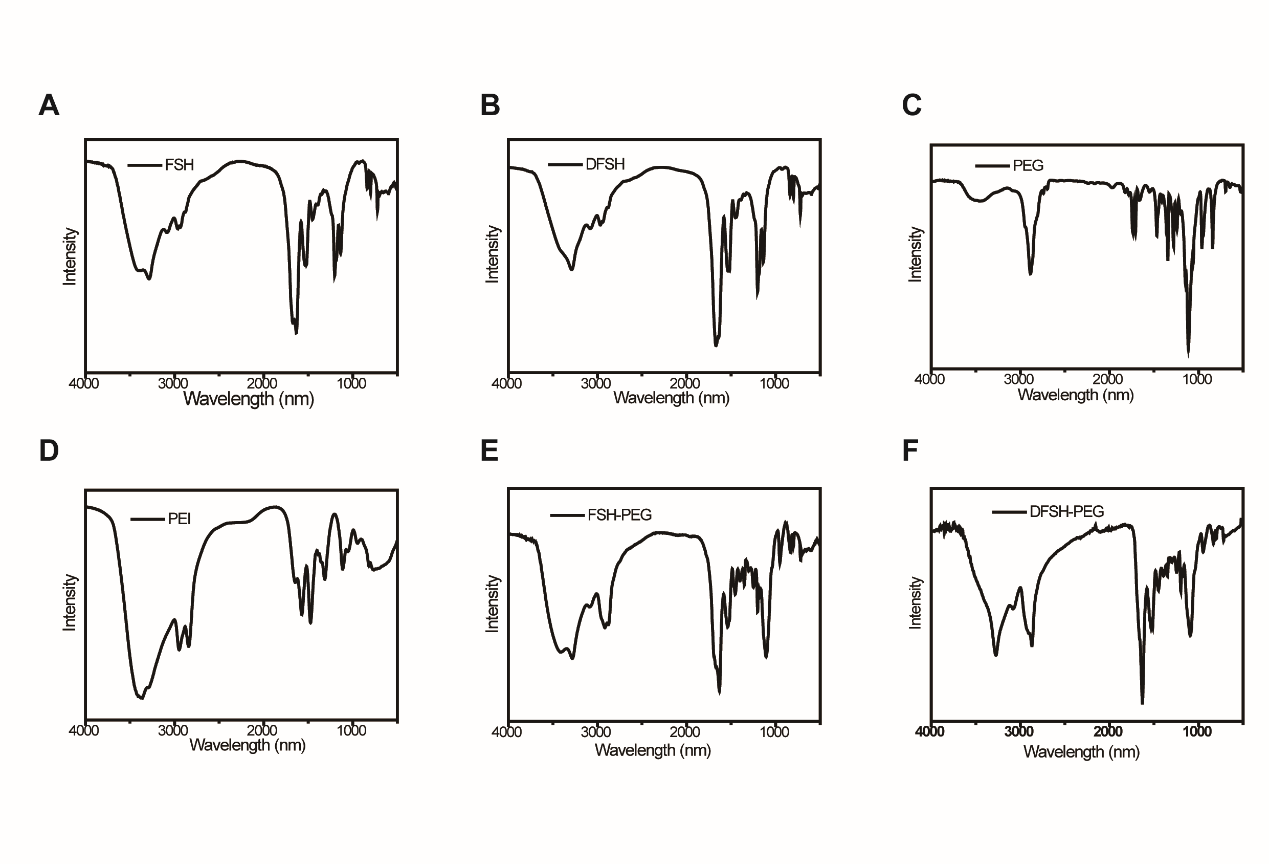
**Figure S2. FTIR of FSH (A), DFSH (B), PEG (C), PEI (D), FSH-PEG (E) and DFSH-PEG (F).**


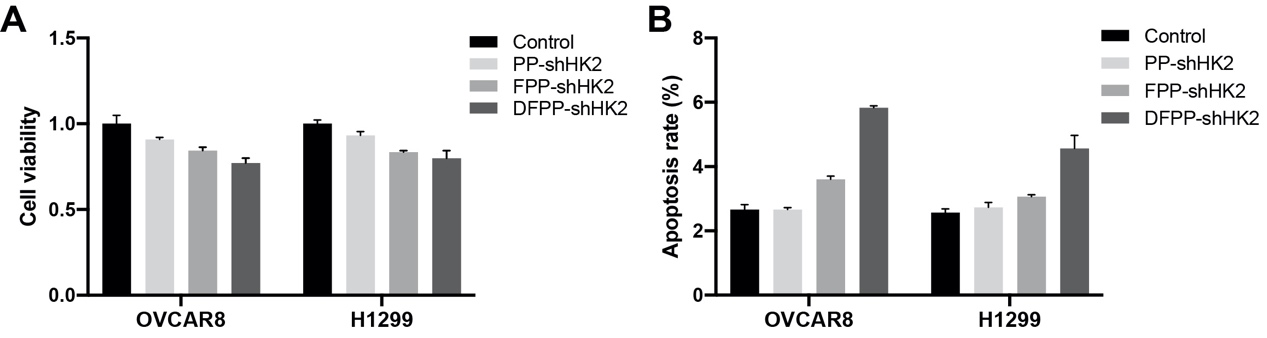


**Figure S3. In vitro effects of HK2 shRNA-loaded nanoparticles on different cancer cell lines.** (A) Cell viability according to a CCK-8 assay. (B) Cell apoptosis according to flow cytometry.


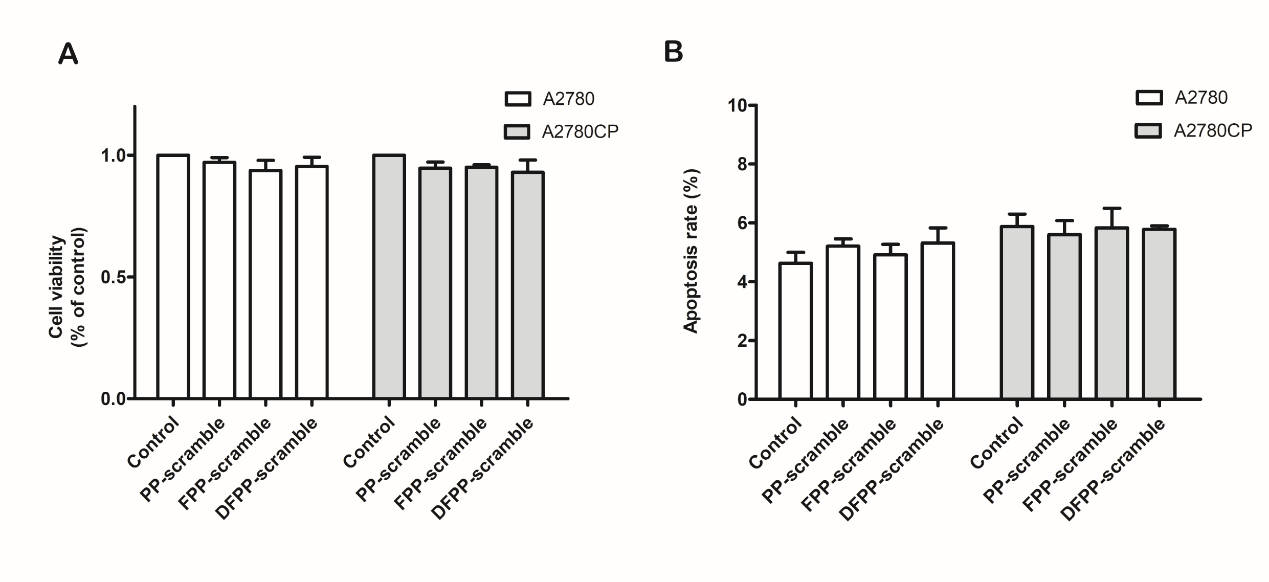
**Figure S4. In vitro effects of scramble shRNA-loaded nanoparticles on A2780 and A2780CP cells.** A2780 and A2780CP cells were treated with scramble shRNA-loaded nanoparticles at a plasmid-equivalent concentration of 1.0 μg/ml. (A) Cell viability according to a CCK-8 assay. (B) Cell apoptosis according to flow cytometry.
